# Dynamic transcriptome analysis unravels key regulatory genes of maize root growth and development in response to potassium deficiency

**DOI:** 10.1007/s00425-023-04260-7

**Published:** 2023-10-14

**Authors:** Song Guo, Zhigang Liu, Huajin Sheng, Toluwase Olukayode, Zijun Zhou, Yonghong Liu, Meng Wang, Mingjiang He, Leon Kochian, Yusheng Qin

**Affiliations:** 1 Institute of Agricultural Resources and Environment, Sichuan Academy of Agricultural Sciences, Chengdu, 610066 People’s Republic of China; 2 Global Institute for Food Security, University of Saskatchewan, Saskatoon, SK S7N 4L8 Canada; 3 Crop Research Institute, Sichuan Academy of Agricultural Sciences, Chengdu, 610066 People’s Republic of China; 4 Institute of Agricultural Resource and Environment, Jilin Academy of Agricultural Sciences, Changchun, 130033 People’s Republic of China

**Keywords:** Differentially expressed genes, Hub genes, Potassium deficiency, Root system architecture, Weighted gene co-expression network

## Abstract

**Main conclusion:**

**Integrated root phenotypes and transcriptome analysis have revealed key candidate genes responsible for maize root growth and development in potassium deficiency.**

**Abstract:**

Potassium (K) is a vital macronutrient for plant growth, but our understanding of its regulatory mechanisms in maize root system architecture (RSA) and K^+^ uptake remains limited. To address this, we conducted hydroponic and field trials at different growth stages. K^+^ deficiency significantly inhibited maize root growth, with metrics like total root length, primary root length, width and maximum root number reduced by 50% to 80% during early seedling stages. In the field, RSA traits exhibited maximum values at the silking stage but continued to decline thereafter. Furthermore, K deprivation had a pronounced negative impact on root morphology and RSA growth and grain yield. RNA-Seq analysis identified 5972 differentially expressed genes (DEGs), including 17 associated with K^+^ signaling, transcription factors, and transporters. Weighted gene co-expression network analysis revealed 23 co-expressed modules, with enrichment of transcription factors at different developmental stages under K deficiency. Several DEGs and transcription factors were predicted as potential candidate genes responsible for maize root growth and development. Interestingly, some of these genes exhibited homology to well-known regulators of root architecture or development in Arabidopsis, such as Zm00001d014467 (*AtRCI3*), Zm00001d011237 (*AtWRKY9*), and Zm00001d030862 (*AtAP2/ERF*). Identifying these key genes helps to provide a deeper understanding of the molecular mechanisms governing maize root growth and development under nutrient deficient conditions offering potential benefits for enhancing maize production and improving stress resistance through targeted manipulation of RSA traits in modern breeding efforts.

**Supplementary Information:**

The online version contains supplementary material available at 10.1007/s00425-023-04260-7.

## Introduction

Potassium (K) is an essential macronutrient for plant growth and development, and its role in various critical physiological processes in plants cannot be overstated. K plays a fundamental role in plant growth and development by enhancing photosynthetic assimilation (Tränkner et al. 2018), improving nutrient uptake (Sustr et al. 2019; Xu et al. 2020), maintaining adequate leaf inclination (Carroll et al. 1994), controlling stomata-opening (Andrés et al. 2014), facilitating chloroplast integrity and light-absorption efficiency, regulating rubisco biosynthesis and activity (Jia et al. 2008; Jákli et al. 2017). It is well-established in regulating plant water balance and ion homeostasis (Schachtman and Shin 2007). Overall, K^+^ plays a crucial part in regulating transport of water, metabolites, and nutrients throughout different plant organs and tissues, as well as in protecting plants from oxidative stress while maintaining osmotic balance.

Although grain crop production has significantly improved over the past few decades, the challenge of meeting global food demands remains significant, particularly due to the persistent problem of inadequate access to energy and protein in the diets of more than 10% of the world’s population (Ray et al. 2013; Grote et al. 2021). Maize is an important cereal crop cultivated extensively for food and feed production (Ranum et al. 2014). It is a critical food crop that plays a significant role in global food security, providing 20% of the world's caloric and 15% of the protein intake (Bhatnagar et al. 2004). For optimal growth and productivity, maize requires high levels of K (Kogbe and Adediran 2003). Nevertheless, K deficiency is a pervasive issue in several maize-growing regions, leading to a significant reduction in crop yield and quality (Wu et al. 2014; Ul-Allah et al. 2020). Hence, comprehending the mechanisms of K uptake, transport, and utilization in maize is crucial for enhancing crop performance and sustainability.

Despite the importance of K in maize nutrition, relatively little is known about the underlying physiological and molecular mechanisms that control K uptake and allocation in different tissues and developmental stages. In this study, we aimed to investigate the performance of maize cultivar CD30 under low K conditions and to identify the key genes and pathways involved in response to K deficiency. Specifically, we addressed two main research questions: (i) How does the root system architecture of maize respond to K deprivation at different growth stages? (ii) What are the transcriptomic changes in maize roots under low K stress, and what are the hub genes and pathways involved in post-transcriptional modulation of the response to K stress? K^+^ plays a critical role in various levels of root system growth and development, including protein synthesis, enzyme activity, cell expansion, root maturation, and phloem transport (Zhao et al. 2016; Zhang et al. 2020; Feng et al. 2021). Root system architecture and root hair coverage can adapt to enhance K^+^ uptake under K limiting conditions, but the molecular mechanism governing this phenomenon in maize remains elusive. Recently, the transcriptome profiles of plant roots under K deficiency have been analyzed in different crops, including rice, wheat, soybean, cotton, and tomato (Ruan et al. 2015; Zhao et al. 2018; Ma et al. 2020; Yang et al. 2021). These analyses have highlighted the crucial role of metabolic pathways and regulatory genes in maintaining plant growth under low K conditions. Additionally, the significance of the root as the primary site for mineral nutrient uptake was emphasized, with a particular emphasis on its role in K uptake regulation. Regulators such as high-affinity K^+^ transporters regulate of root K uptake in response to deficiency, further underscoring the root's importance (Sustr et al. 2019). Recently, a study explored the growth, transcriptional, and metabolic responses of maize shoots to long-term potassium deficiency. They found that under K insufficiency conditions, the biomass yield of silage maize decreased, and several genes were differentially regulated, particularly many stress-induced genes (Xiong et al. 2022). Given the importance of K deficiency in several aspects of plant growth, it is essential to understand how root architecture is modulated under inadequate K nutrition.

To answer these questions, we conducted a series of experiments to evaluate the effects of K deficiency on maize growth, biomass accumulation, root system architecture (RSA), and grain yield, as well as the changes in gene expression and regulation at five key developmental stages. We used a hydroponic pouch system to simulate K deprivation in early seedling stages and conducted field trials to assess the phenotypic traits and nutrient concentrations at different growth stages. We also performed transcriptomic analysis using RNA sequencing (RNA-seq) to identify differentially expressed genes (DEGs) and co-expression modules in response to K stress and to identify the hub genes and pathways involved in post-transcriptional modulation of the response to K stress.

## Materials and methods

### Plant material

We used *Zea mays* cv. Chengdan 30 (CD30), a high-yielding spring maize cultivar developed by the Sichuan Academy of Agricultural Sciences in 2004 and being currently dominant in the hilly region of central Sichuan (Guo et al. 2022).

### Assessment of root system architecture (RSA) in hydroponics

Maize seeds were surface-sterilized with 8% (v/v) sodium hypochlorite for 20 min. The sodium hypochlorite was removed by washing the seeds with distilled water three times. The seeds were then germinated in moistened filter paper. Four days after germination, seedlings with similar growth vigor were transferred to 100 L polypropylene tubs and grown hydroponically using a specially designed and constructed plant growth pouch system, which holds the seedling and root system on the surface of filter paper, as described by Gladman et al. (2022). The full strength nutrient solution contains: 1 mM Ca(NO_3_)_2_·4H_2_O, 1 mM NH_4_NO_3_, 1 mM KCl, 0.85 mM MgSO_4_·7H_2_O, 0.25 mM NH_4_H_2_PO_4_, 77 μM Fe-EDTA, 25 μM H_3_BO_3_, 0.8 μM Na_2_MoO_4_·2H_2_O, 0.6 μM CuSO_4_·5H_2_O, 9 μM MnCl_2_·4H_2_O, 2 μM ZnSO_4_·7H_2_O. For a nutrient solution with low-K (LK) treatment, the potassium concentration was adjusted to 10 μM and 1000 μM as sufficient K conditions (SK), respectively. The pH of the solution was adjusted to 5.7 ± 0.1 with NaOH or HCl as required and was continuously aerated and replaced every 3 days. The seedlings were cultured in a growth chamber (16 h of light at 27 °C, 8 h dark at 22 °C, a photosynthetic photon-flux density of ~ 350 µmol m^−2^ s^−1^ at canopy height, and 60% relative humidity). Each treatment had six replicates.

After 14 days of growth in hydroponics, individual pouches with one maize plant were removed from the hydroponic tub. The plant on the pouch system was carefully placed horizontally in a glass tray containing water, and the root system was submerged in the water to collect root system images using Plant Root Imaging and Data Acquisition (PRIDA), a Python-based image acquisition and data management software (http://www.plantmineralnutrition.net/software/prida/index.html). The images were extracted as TIFF files for further processing and computation of root traits. WinRHIZO software (Regents Instruments Inc, Quebec, Canada) was used to quantify root growth and topology traits, while GiA Roots (Galkovskyi et al. 2012) was used to quantify 2D root architecture traits. The investigated root architecture traits included: total root length (cm), root surface area (cm^2^), primary root length (cm), root width (cm), average root diameter (cm), convex area (cm^2^), maximum root number, and root volume (cm^3^). After imaging, the whole maize seedlings were harvested, and the shoots and roots were dissected. The samples were heated at 105 ℃ for 30 min and then oven-dried at 65 ℃ for 72 h. The shoot dry weight, root dry weight, and root-to-shoot ratio (R/S) were determined by weighing the samples.

### Field experiment, sample collection and phenotype investigation

A field experiment was conducted at Sichuan Agricultural Research Institute Modern Experimental Station, Deyang City, Sichuan Province. The experimental design was a spit-plot with potassium (K) fertilizer treatments in the main plots and the cultivar in the subplots. The variable between two application rates of K fertilizer, namely 150 kg K ha^−1^ (SK) and no K supplied (LK). No K fertilizer has been applied since 2012. The plots were fertilized with 300 kg ha^−1^ N and 90 kg ha^−1^ P_2_O_5_. Phosphorus and K fertilizers were applied before sowing, 50% of N fertilizer was applied as a base dressing, and the remaining 50% was applied at the stem jointing stage. Soil physical and chemical characteristics were evaluated at the beginning of the experiment for SK and LK treatments by analyzing three soil samples, respectively (Suppl. Table S1). The topsoil layer (0–30 cm) in SK treatment contained organic matter 11.4 g kg^−1^, total N 0.83 g kg^−1^, alkali-hydrolyzable N 63 mg kg^−1^, available phosphorus (Olsen-P) 5.9 mg kg^−1^, ammonium acetate extractable potassium 133 mg kg^−1^ and pH 8.60 (1:1.25 g/v). In LK treatment, chemical characteristics were organic matter 9.9 g kg^−1^, total N 0.76 g kg^−1^, alkali-hydrolyzable N 55 mg kg^−1^, available phosphorus (Olsen-P) 5.7 mg kg^−1^, ammonium acetate extractable potassium 93 mg kg^−1^ and pH 8.61. The subplot area was 20 m^2^ (5-m-long × 4-m-width). Maize was seeded on 1st April and harvested on 11th August 2021.

During specific developmental stages, namely the 10th leaf stage (V10), tasseling stage (VT), silking stage (R1), milk-ripe stage (R3), and physiological maturity (R6), root samples were obtained for RNA sequencing. We selected three uniform phenotype plants for each stage, carefully extracting two layers of roots from beneath the soil surface. These root samples were subsequently cleaned using running water and combined into a single representative sample. To ensure robust analysis, we collected three replicates for each developmental stage, promptly freezing them in liquid nitrogen, and then storing them at − 80 °C for subsequent RNA isolation.

Total RNA was isolated from root tissues using a Trizol reagent (Invitrogen, Carlsbad, CA, USA) following the manufacturer’s instructions. RNA integrity was confirmed using the 2100 Bioanalyzer system (Agilent Technologies, Santa Clara, CA, USA). Reverse transcription was performed to synthesize first-strand cDNAs as templates for preparing mRNA-seq libraries. All RNA-seq libraries were constructed using a Ribo-Zero rRNA Removal kit (Illumina Inc., San Diego, CA, USA) followed by a TruSeq Stranded Total RNA Library Prep Plant and then sequenced on an Illumina HiSeq2500 Platform (NovoMagic, Beijing, China).

Root system architecture (RSA) was evaluated at each developmental stage. Three uniform phenotype plants were selected from the central rows of each subplot, then the soil volume with 33 cm in width, 50 cm in length and 30 cm in depth surrounding the plant was excavated, and the whole seedlings were dissected into shoot and root. The roots were cleaned with running water. Root segments were floated in water in an acrylic tray on the scanner (ESPON Perfection V850). The scanned images were analyzed with WinRHIZO (Regents Instruments Inc.) commercial software to obtain total root length, surface area, volume and average root diameter. After imaging, the shoot and root were heated at 105 ℃ for 30 min and then oven dried at 65 °C for 72 h to obtain shoot and root dry weight. The grain yield was determined and then standardized to 14% moisture. Total N concentration in grain and plant shoot was determined by the semi-micro Kjeldahl procedure (Bremner 1996), total P concentration by spectrophotometer (Chapman and Pratt 1962) and total K concentration by flame photometer (Chapman and Pratt 1962), respectively.

### Alignment and identification of differentially expressed genes

The RNA-seq reads generated by the Illumina Genome Analyzer were initially preprocessed to remove low-quality reads and adaptor sequences. After filtering, the clean reads of each sample were used to align the reads to the maize reference genome (B73 RefGen_V4, http://ensemblgramene.org/Zea_mays/Info/Index) using Hisat2 (Kim et al. 2015). Differentially expressed genes (DEG) were identified by the DESeq2 package in R (Love et al. 2014) with the absolute value of the Log_2_ fold change ≥ 1.0, *P* value < 0.05 (Luo et al. 2021; Gao et al. 2022) between LK and SK conditions at each growth stage. The fragments per kilobase of transcript per million reads (FPKM) of each gene were calculated for further use.

### Functional enrichment analysis

To determine which DEGs were significantly enriched in Gene Ontology (GO) terms at a Bonferroni-corrected *P* value < 0.05 compared to the whole-transcriptome background, we conducted GO enrichment analysis. The GO functional enrichment analysis was performed using agriGO v2.0 (http://systemsbiology.cau.edu.cn/agriGOv2/index.php) with singular enrichment analysis (Du et al. 2010). Kyoto Encyclopedia of Genes and Genomes (KEGG) pathway annotation and enrichment analysis of the DEGs was performed using the website https://www.genome.jp/kegg/, with a corrected *P* value of 0.05 as the threshold for significantly enriched KEGG.

### Weighted gene co-expression network analysis (WGCNA)

The FPKM value of each gene from the five comparisons (i.e., V10, VT, R1, R3, and R6) was used for co-expression analysis using the WGCNA (Langfelder and Horvath 2008). The soft threshold was determined whereby more than 85% of the models would fit to scale-free topology and low mean connectivity. The minimum gene number assigned to one module was set as 30. The top 20 connected hub genes in each module were used to construct the module network graphs using Cytoscape software (version 3.7.1).

### Statistical analysis

We performed all statistical analyses using the R software package (Team 2013). To test for significant differences between treatments, we used a one-way analysis of variance (ANOVA), which was conducted using the “Anova” function as implemented in the “car” package (Fox et al. 2012). The data were visualized using the “ggplot2” package (Wickham 2016).

## Results

### Performance of root system architecture response to low K

**Fig. 1:**
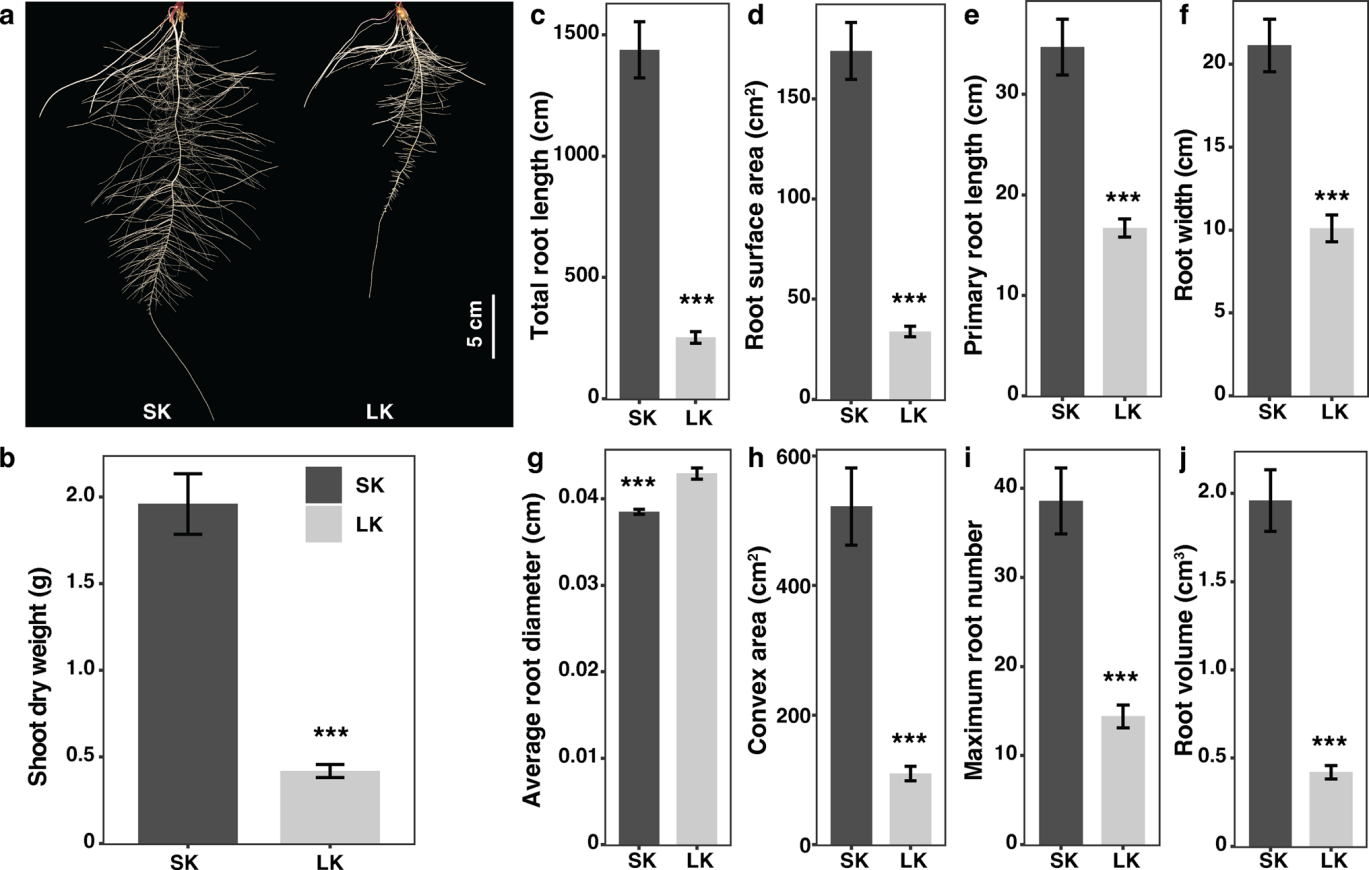
Root system architecture of maize cultivar grown under sufficient K (SK) and low K (LK) conditions. Plants were grown in a hydroponic pouch system and harvested at 14 days after transplanting. **a** Representative root images of CD30 under SK and LK conditions, **b** Shoot dry weight, **c** total root length, **d** root surface area, **e** primary root length, **f** root width, **g** average root diameter, **h** convex area, **i** maximum root number, and **j** root volume under SK and LK conditions. Mean values ± SE, *n* = 6. Asterisks indicate the significance of differences between treatments, as determined by Student’s t test: *Significant at *P* < *0.05*; **Significant at *P* < *0.01*; ***Significant at *P* < *0.001*

**Fig. 2:**
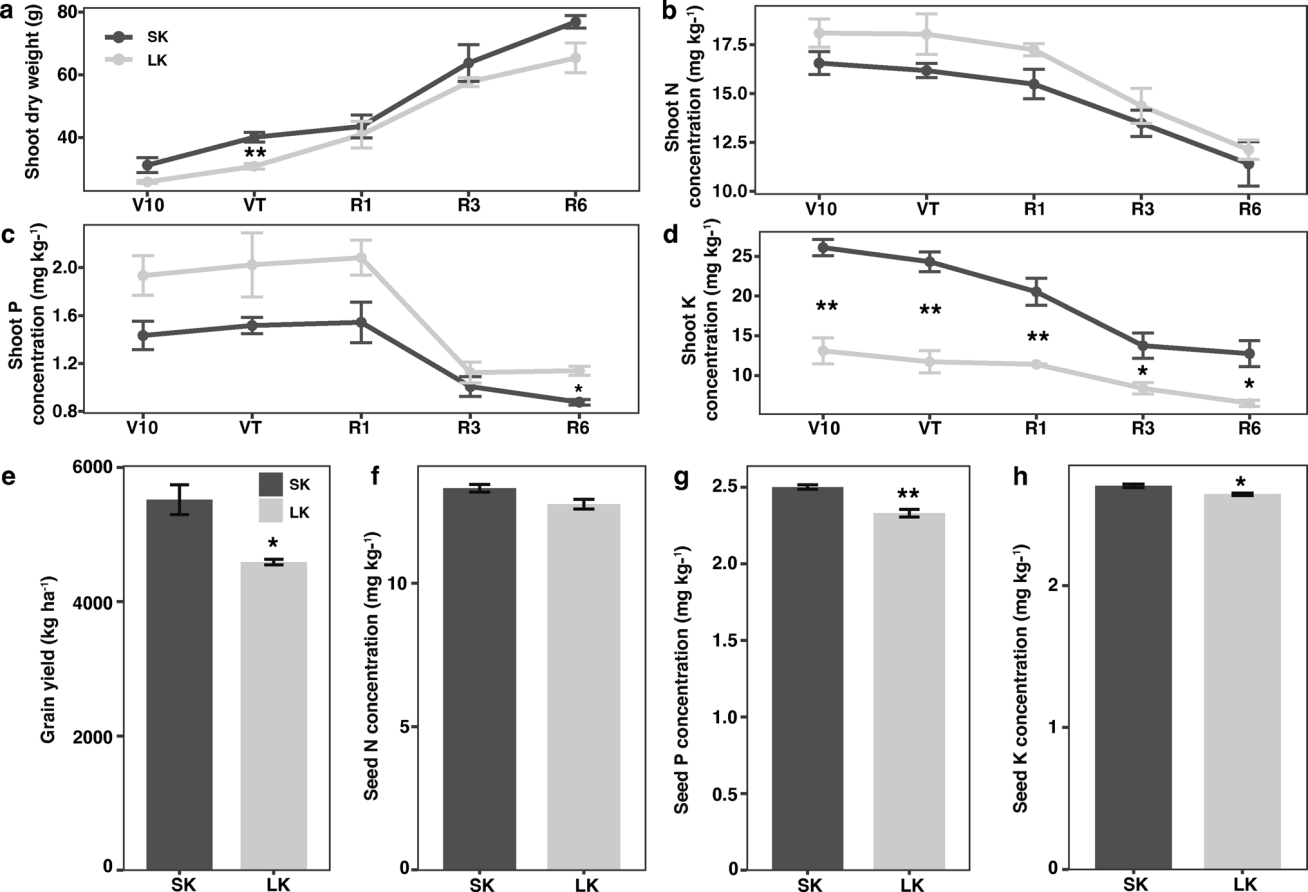
Phenotypic identification of maize cultivar in the field. **a** Shoot dry weight, **b** shoot N concentration, **c** shoot P root dry weight concentration, **d** shoot K concentration, **e** grain yield, **f** seed N concentration, **g** seed P concentration, and **h** seed K concentration at five different developmental stages under sufficient K (SK) and low K (LK) conditions. Mean values ± SE, *n* = 3. Asterisks indicate the significance of differences between treatments, as determined by Student’s t test: *Significant at *P* < *0.05*; **Significant at *P* < *0.01*; ***Significant at *P* < *0.001.* V10: the 10th leaf stage, *VT* tasseling stage, *R1* silking stage, *R3* milk-ripe stage, *R6* physiological maturity

We evaluated maize cultivar CD30 performance of the above-ground and below-ground parts at early seedling and late developmental stages in response to potassium (K) deficit (Figs. 1 and 2). In the hydroponic pouch system, K deprivation reduced shoot dry weight by 67.7% (Fig. 1b). Significant differences were also observed in RSA between sufficient K (SK) and low K (LK) treatments (Fig. 1a). Total root length, primary root length, root width, and maximum root number were reduced under K starvation by 82.4%, 51.9%, 52.2%, and 62.7%, respectively. A similar trend was observed for root surface area, convex area, and root volume. The average root diameter increased by 11.5% under LK conditions (Fig. 1c–j).

**Fig. 3:**
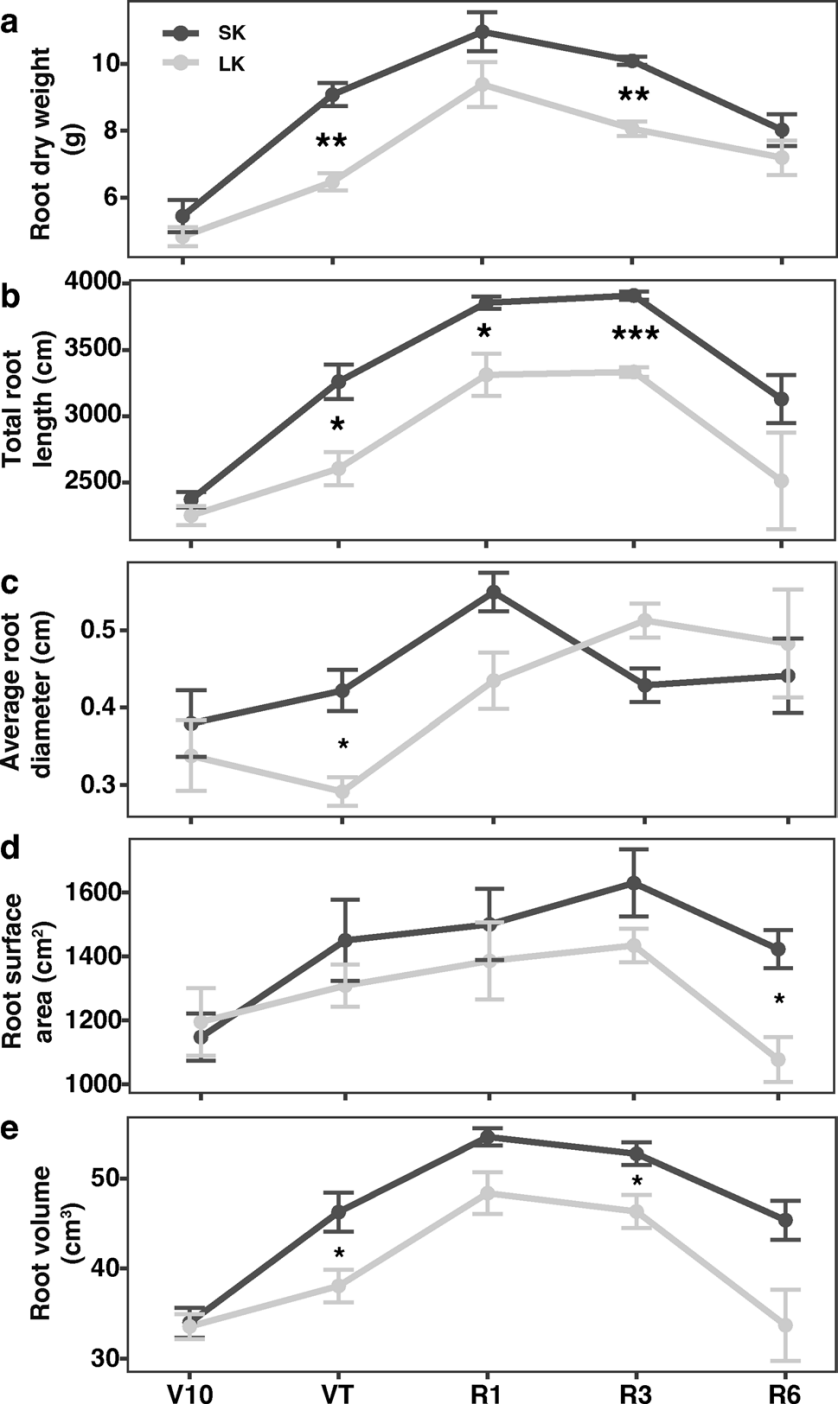
Root system characterization in field conditions. **a** Root dry weight, **b** total root length, **c** average root diameter, **d** root surface area, and **e** root volume at five different developmental stages under sufficient K (SK) and low K (LK) conditions. Mean values ± SE, *n* = 3. Asterisks indicate the significance of differences between treatments, as determined by Student’s t test: *Significant at *P* < *0.05*; **Significant at *P* < *0.01*; ***Significant at *P* < *0.001.* *V10* the 10th leaf stage, *VT* tasseling stage, *R1* silking stage, *R3* milk-ripe stage, *R6* physiological maturity

In the field, phenotypic identification of RSA and biomass-related traits was carried out at V10, VT, R1, R3, and R6 stages, respectively. Grain yield and N, P, and K concentrations in seeds were also investigated at the mature stage. Shoot dry weight in SK is slightly higher than in LK condition at different developmental stages. Only a significant difference was observed at VT (Fig. 2a). However, K concentration in the shoot was reduced significantly in response to LK starvation at all tested stages. K deficit may not affect N and P concentration in the shoot part (Fig. 2b–d). K starvation led to a 16.8% (*P* < *0.05*) decrease in grain yield, a 2.2% (*P* < *0.05*) decrease in seed K concentration, and a 6.8% (*P* < *0.01*) in seed P concentration, respectively, but not altered seed N concentration (Fig. 2e–h). Root dry weight was increased from V10 to VT in both conditions, reached the maximum values at R1 stages and then continued decline. Significant differences in root dry weight were observed at VT and R3 stages between SK and LK conditions (Fig. 3a). A similar pattern was observed in RSA traits, where SK showed an average larger total root length (16.3%), root surface area (10.3%), and root volume (14.7%) than in LK conditions (Fig. 3b–d).

### Transcriptomic of maize root response to low K stress

We performed transcriptomic analysis of maize roots by RNA-seq at five key developmental stages (V10, VT, R1, R3, and R6). To mitigate background influences, the roots from three plants were pooled as one sample. Thirty RNA-seq libraries were constructed and sequenced from maize roots at different developmental stages with three biological replicated under SK and LK conditions. Totally, 142 million raw reads were generated for all the samples, and 138 million clean reads (97.27%) were obtained after performing quality control with a Q20 value of 97.68%. The average GC content is 54.83% (Suppl. Table S2). Then, the clean reads were aligned against the maize reference genome (B73 RefGen_V4), and an average of 82.43% clean reads were mapped to the B73 reference genome (Suppl. Table S3). A 77.72% of known genes (30,565 known genes *vs*. 39,324 reference genes) were covered, and an average of 4,811 new genes were detected among the five developmental stages. The detected known gene number of each stage were 31,276 (LK) and 31,063 (SK) for V10, 30,673 (LK) and 31,137 (SK) for VT, 30,783 (LK) and 30,221 (SK) for R1, 30,071 (LK) and 30,056 (SK) for R3, 30,124 (LK) and 30,294 (SK) for R6 (Suppl. Table S4) The Pearson correlation analysis plots showed strong correlation coefficient among the replicates under SK and LK conditions, respectively, indicating the reliability of our RNA-seq data (Suppl. Fig. S1).

**Fig. 4:**
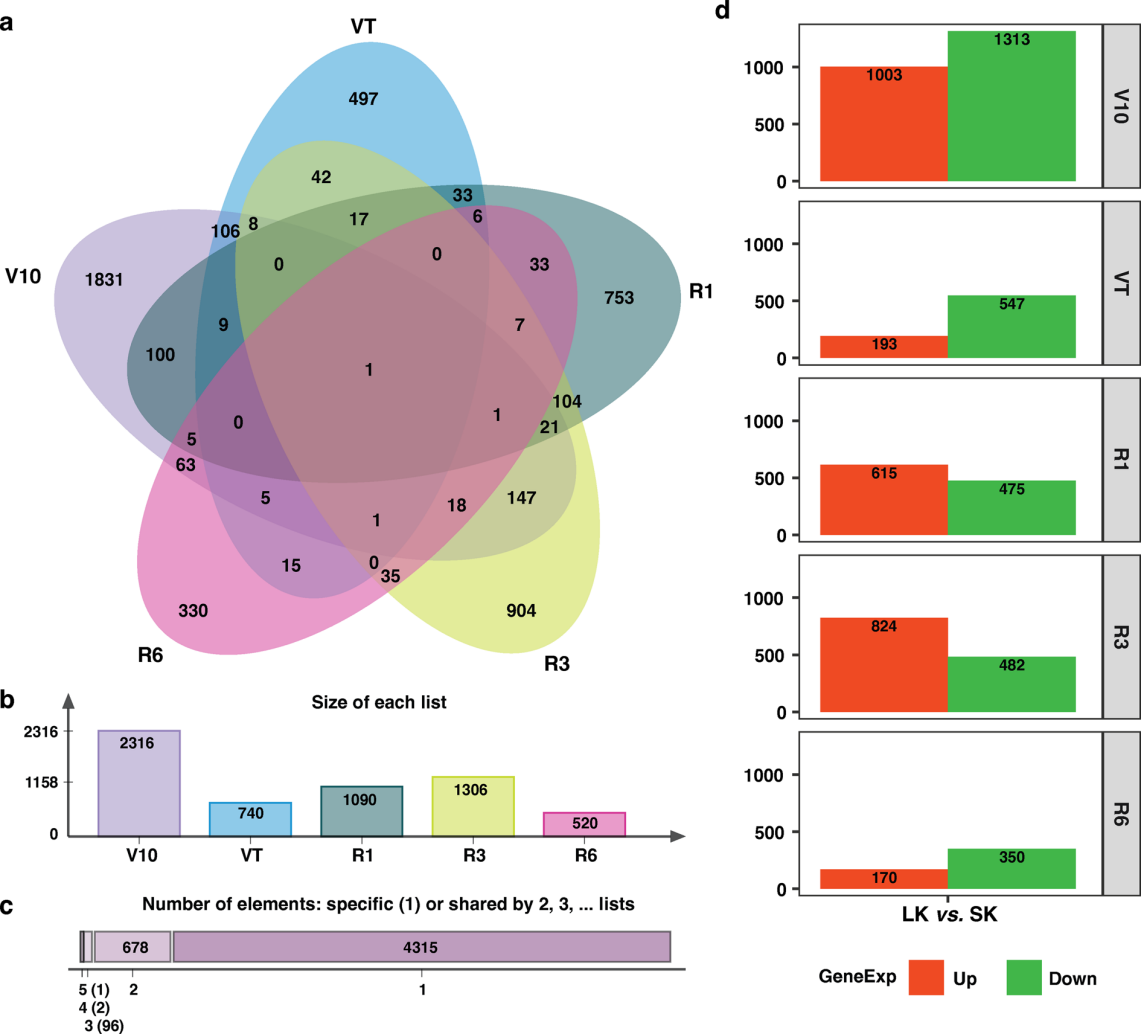
Differentially expressed genes (DEGs) analysis in maize cultivar CD30 in response to K stress. **a** Venn diagram of DEGs, **b** DEGs number in each developmental stage, **c** Shared DEGs number among the five different stages, **d** Summary of up-regulated and down-regulated DEGs number in each developmental stage. *V10*: the 10th leaf stage, *VT* tasseling stage, *R1* silking stage, *R3* milk-ripe stage, *R6* physiological maturity

We performed a pairwise comparison between SK and LK conditions at five developmental stages. Totally, 5,972 genes were referred to as DEGs and used for the subsequent analysis. The number of DEGs at each stage was as follows: 2,316 (V10), 740 (VT), 1,090 (R1), 1,306 (R3), and 520 (R6), respectively. Of those DEGs, 4,315 specific genes were identified at all stages, 678 genes were shared by two development stages, 93 genes by three, two genes by four, and one gene by five stages, respectively (Fig. 4a–c). To better understand the dynamic changes of gene expression in maize roots with all the five developmental stages, further analyses of the DEGs were performed, especially of those genes in which up- or down-regulated follow root development. Within the identified DEGs, 1,003, 193, 615, 824, and 170 DEGs were up-regulated in stages V10, VT, R1, R3 and R6. Likewise, 1,313 (V10), 547 (VT), 475 (R1), 482 (R3), and 350 (R6) DEGs were down-regulated at each developmental stage (Fig. 4d).

Additionally, many genes related to the K^+^ signaling pathway, transcription factors and transporters were identified (Table 1). For instance, a type III peroxidase family, *Rare Cold Inducible gene 3* (*RCI3*), was a component of the low K signal transduction pathway in Arabidopsis roots. It was reported that *RCI3* was induced upon K deprivation and participated in the induction of the high-affinity K^+^ uptake transporter *HAK5*, which ultimately facilitated K acquisition under K starvation (Kim et al. 2010). In maize roots, Zm00001d014467, the homolog gene of *RCI3,* was up-regulated in response to LK condition in V10 and R1 stages. Several other K signaling genes such as *SYNTAXIN OF PLANTS 121* (Zm00001d013114), *CALCINEURIN B-LIKE INTERACTING PROTEIN KINASE 23* (Zm00001d018799), *CALCINEURIN B-LIKE PROTEIN 10* (Zm00001d044285) were also observed up-regulated in specific developmental stage under LK conditions. Moreover, important transcription factors (TFs) related to K metabolism were found on the list. Maize *WRKY TRANSCRIPTION FACTOR 9* (Zm00001d011237, Zm00001d018203) was significantly induced by LK in VT, R3, and R6 stages (Table 1). In Arabidopsis, *RAP2.11* is an *AP2/ERF* transcription factor, identified as a component in response to low K affecting root hair development (Kim et al. 2012). The homolog gene in maize, Zm00001d030862, was observed down-regulated in root tissue at the earlier developmental stage. Remarkably, the homolog gene of high-affinity K^+^ uptake transporter *HAK5* in maize, Zm00001d033067, down-regulated in root tissue at the V10 stage and up-regulated upon K deprivation at later developmental stages. The transporter *POTASSIUM CHANNEL PROTEIN* (Zm00001d016160) was notably induced in response to K starvation at V10, VT, and R1 stages, respectively (Table 1).

**Table 1:** Genes involved in K-related pathways in this study

Annotation	Gene ID	Log2FC					Closet AGI	Symbol of AGI	Description	References
		V10	VT	R1	R3	R6				
Signaling	Zm00001d013114	–	–	–	1.4	–	AT3G11820	SYP121	SYNTAXIN OF PLANTS 121	(Schachtman et al. 1992)
	Zm00001d014467	1.7	–	5.0	–	–	AT1G05260	RCI3	RARE COLD INDUCIBLE GENE 3	(Hong et al. 2013)
	Zm00001d018799	–	–	–	1.3	–	AT1G30270	CIPK23	CALCINEURIN B–LIKE INTERACTING PROTEIN KINASE 23	(Ragel et al. 2015)
	Zm00001d038419	–	–	− 2.3	–	–	AT5G02600	NAKR1	HEAVY METAL ASSOCIATED PROTEIN	(Tian et al. 2010)
	Zm00001d044285	–	–	–	1.0	–	AT4G33000	CBL10	CALCINEURIN B–LIKE PROTEIN 10	(Ren et al. 2013)
Transcription factor	Zm00001d011237	–	3.4	–	3.1	1.6	AT1G68150	WRKY9	WRKY TRANSCRIPTION FACTOR 9	(Shin and Schachtman 2004)
	Zm00001d013492	2.2	–	–	–	–	AT5G03280	EIN2	ETHYLENE INSENSITIVE 2	(Schachtman et al. 1992)
	Zm00001d018203	–	–	–	1.7	–	AT1G68150	WRKY9	WRKY TRANSCRIPTION FACTOR 9	(Hong et al. 2013)
	Zm00001d030862	− 1.4	–	–	–	–	AT5G19790	RAP2.11	ERF/AP2 TRANSCRIPTION FACTOR FAMILY (RAP2.11)	(Kim et al. 2012)
Transporter	Zm00001d009295	− 3.2	1.1	–	–	− 2.3	AT4G23640	AKT3	K + TRANSPORTER 3	(Ren et al. 2013)
	Zm00001d010210	1.3	–	–	–	–	AT4G22200	AKT2	K + TRANSPORTER 2	(Gajdanowicz et al. 2011)
	Zm00001d016160	3.1	3.5	2.7	–	–	AT4G18290	KAT2	POTASSIUM CHANNEL PROTEIN	(Hong et al. 2013)
	Zm00001d017666	− 1.3	− 1.1	–	–	1.1	AT1G32450	NRT1.5	NITRATE TRANSPORTER 1.5	(Hong et al. 2013)
	Zm00001d018918	− 1.8	–	–	–	–	AT4G32500	AKT5	K + TRANSPORTER 5	(Pilot et al. 2003)
	Zm00001d031923	–	–	–	–	–	AT5G51710	KEA	K + EFFLUX ANTIPORTER	(Ahn et al. 2004)
	Zm00001d033067	− 2.6	–	2.9	4.0	–	AT4G13420	HAK5	HIGH AFFINITY K + TRANSPORTER 5	(Ragel et al. 2015)
	Zm00001d037289	–	–	4.1	–	–	AT5G37500	GORK	GATED OUTWARDLY-RECTIFYING K + CHANNEL	(Corratge-Faillie et al. 2017)

*AGI* arabidopsis gene ID, *V10* the 10th leaf stage, *VT* tasseling stage, *R1* silking stage, *R3* milk-ripe stage, *R6* physiological maturity

### Co-expression network analysis

**Fig. 5:**
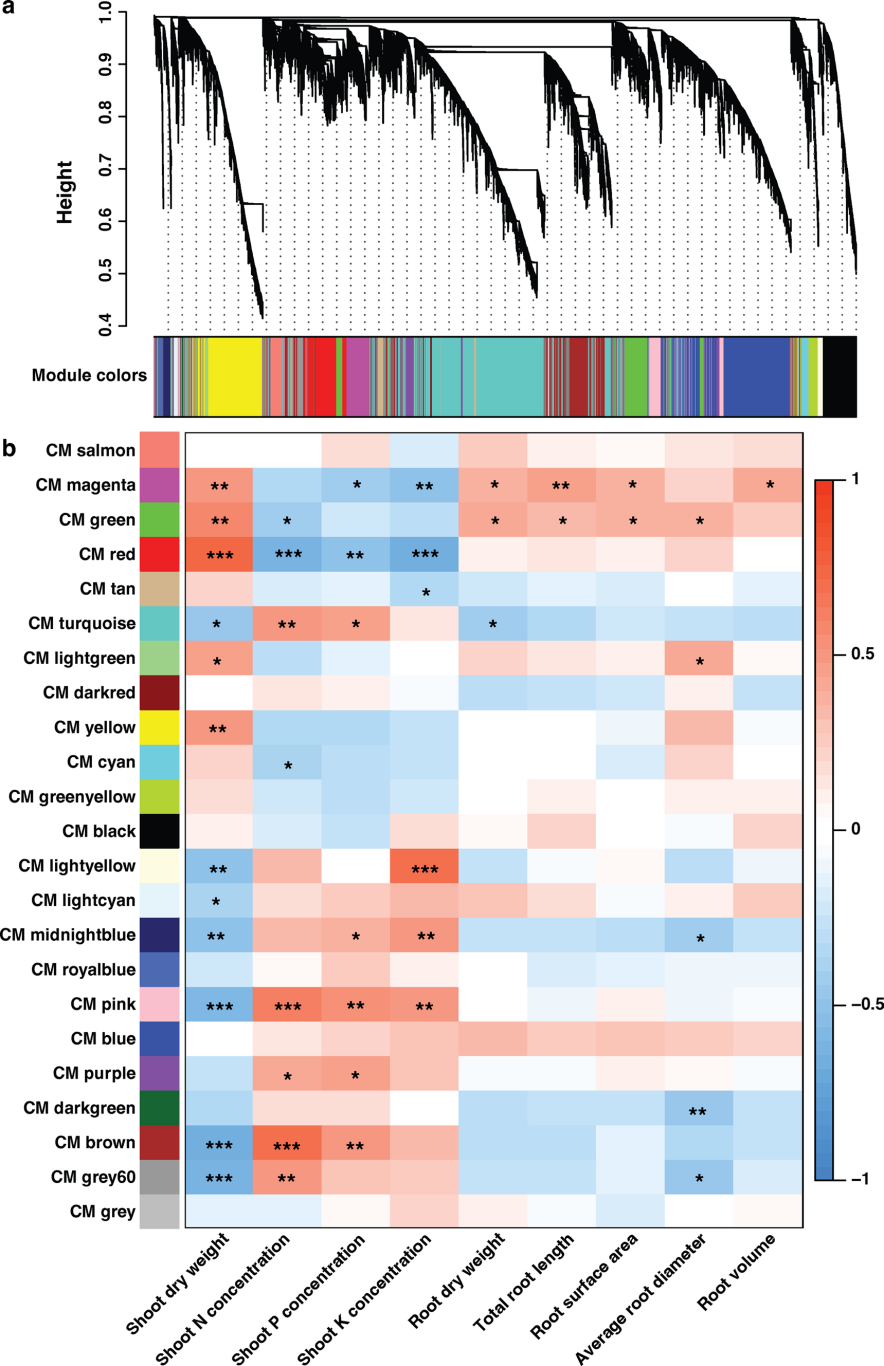
Weighted gene co-expression network analysis (WGCNA). **a** The significantly differentially expressed genes were assigned to different co-expression modules (CMs). CMs were identified via the Dynamic Tree Cut method; the merged dynamic indicates modules divided according to the similarity of the module (represented by assigned module colors). The subsequent analysis was conducted considering the merged modules. In the tree diagram, the vertical distance indicates the separation between two nodes, corresponding to the genetic distance between the genes. **b** The correlative relationships of the assigned CMs and root system architecture. *Significant at *P* < *0.05*; **Significant at *P* < *0.01*; ***Significant at *P* < *0.001*

A gene regulation network was built using the WGCNA method to obtain a comprehensive understanding of the transcriptional network in the successive developmental stages and to clarify that it controls the adaptation of LK condition in maize roots. Totally, 24,043 expressed genes identified in 30 samples were divided into 23 co-expressed modules (CMs), color-coded for reference, with the CM grey set containing the genes that could not be assigned to other CMs (Fig. 5a). The 23 CMs correlated with phenotypic traits are shown in Fig. 5b. A positive correlation was observed in modules of magenta and green with root biomass and RSA traits. There was a positive correlation of magenta (*P* < *0.01*), green (*P* < *0.01*), and red (*P* < *0.001*) CMs with shoot dry weight and a positive correlation of the pink (*P* < *0.001*) module with shoot K concentration. A negative correlation with shoot dry weight was observed in light yellow, midnight blue, and pink CMs.

**Fig. 6:**
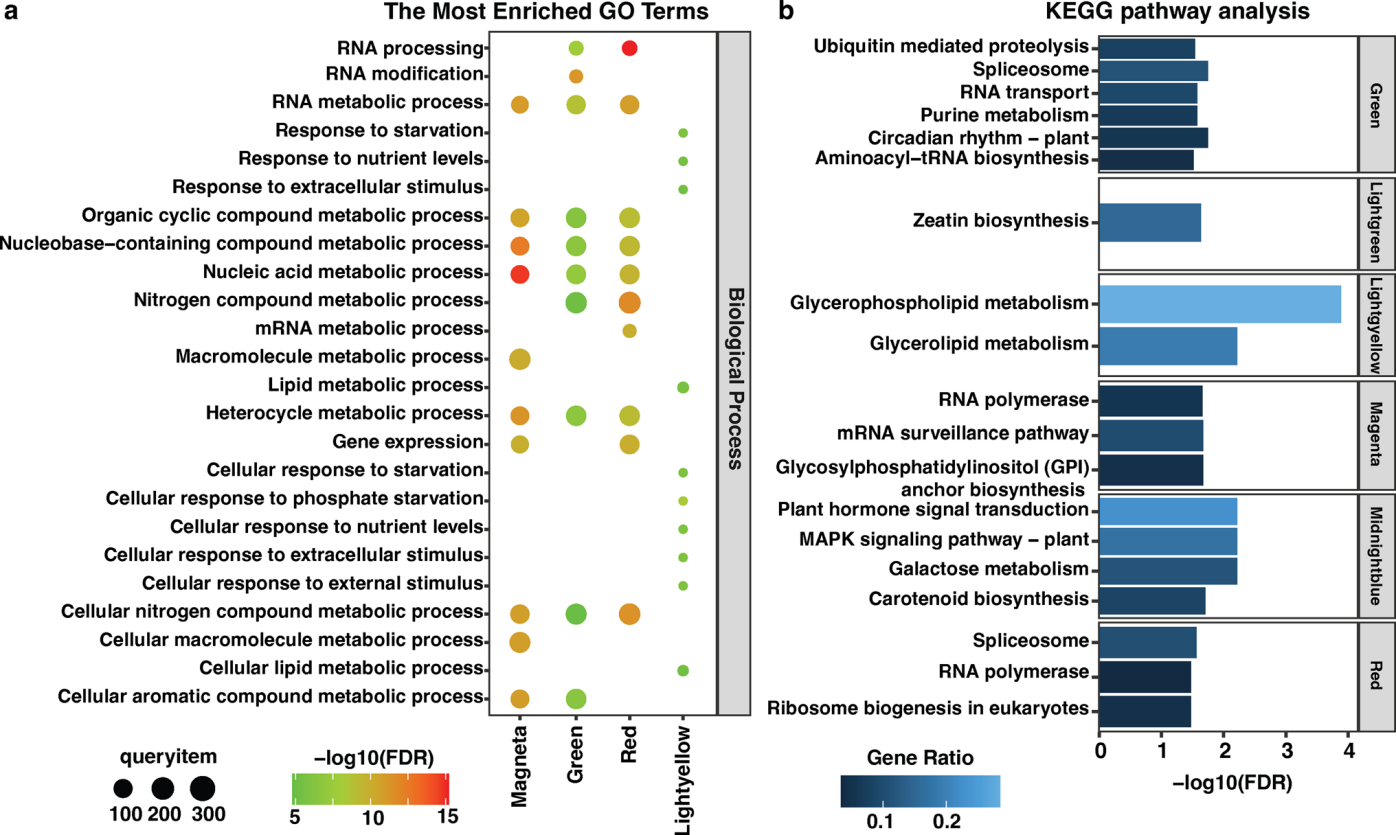
Transcriptomic analysis of different co-expression modules (CMs). **a** The most significant biological process of GO terms for the genes in different CMs. **b** KEGG enrichment analysis in different CMs

We further analyzed the magenta, green, red, light green, light yellow, midnight blue, and pink modules among all K deficit responding modules. Gene ontology enrichment analysis of each selected module highlighted key biological processes by a set of co-expressed genes (Fig. 6a and Suppl. Table S5). The CM magenta (799 genes), associated with shoot dry weight, root dry weight, total root length, root surface area, and root volume traits, showed enrichment of GO terms related to nucleic acid metabolic process, nucleobase-containing compound metabolic process, and regulation of gene expression. A total of 1372 genes grouped into the CM green, and RNA modification, RNA metabolic process, and nitrogen compound metabolic process were enriched in this module. The red module consisted of 1216 genes and showed enrichment of GO terms related to RNA processing, nitrogen compound metabolic process and nucleic acid metabolic process. The CM light yellow identified 157 genes and was mainly involved in the cellular response to starvation and extracellular stimulus. A total of 194, 286, and 971 genes grouped into the light green, midnight blue, and pink modules, respectively. Likewise, the CMs do not show a significant enrichment of GO terms in biological processes (Fig. 6a and Suppl. Table S5).

The DEGs identified in the CM green were found to be significantly enriched in several KEGG pathways, including circadian rhythm—plant (zma04712), spliceosome (zma03040), RNA transport (zma03013), purine metabolism (zma00230), ubiquitin-mediated proteolysis (zma04120), and aminoacyl-tRNA biosynthesis (zma00970). In CM light green, a noteworthy KEGG pathway enriched was zeatin biosynthesis (zma00908). In contrast, two significant KEGG pathways, glycerophospholipid metabolism (zma00564) and glycerolipid metabolism (zma00565), were observed in the CM light yellow. In the CM midnight blue, the DEGs were notably enriched in four KEGG pathways: Galactose metabolism (zma00052), plant hormone signal transduction (zma04075), MAPK signaling pathway—plant (zma04016), and carotenoid biosynthesis (zma00906). In the case of CM magenta and red, the DEGs were significantly enriched in the RNA polymerase (zma03020) pathway (Fig. 6b and Suppl. Table S6).

### Hub genes involved in post-transcriptional modulation are essential regulators in response to K stress

**Fig. 7:**
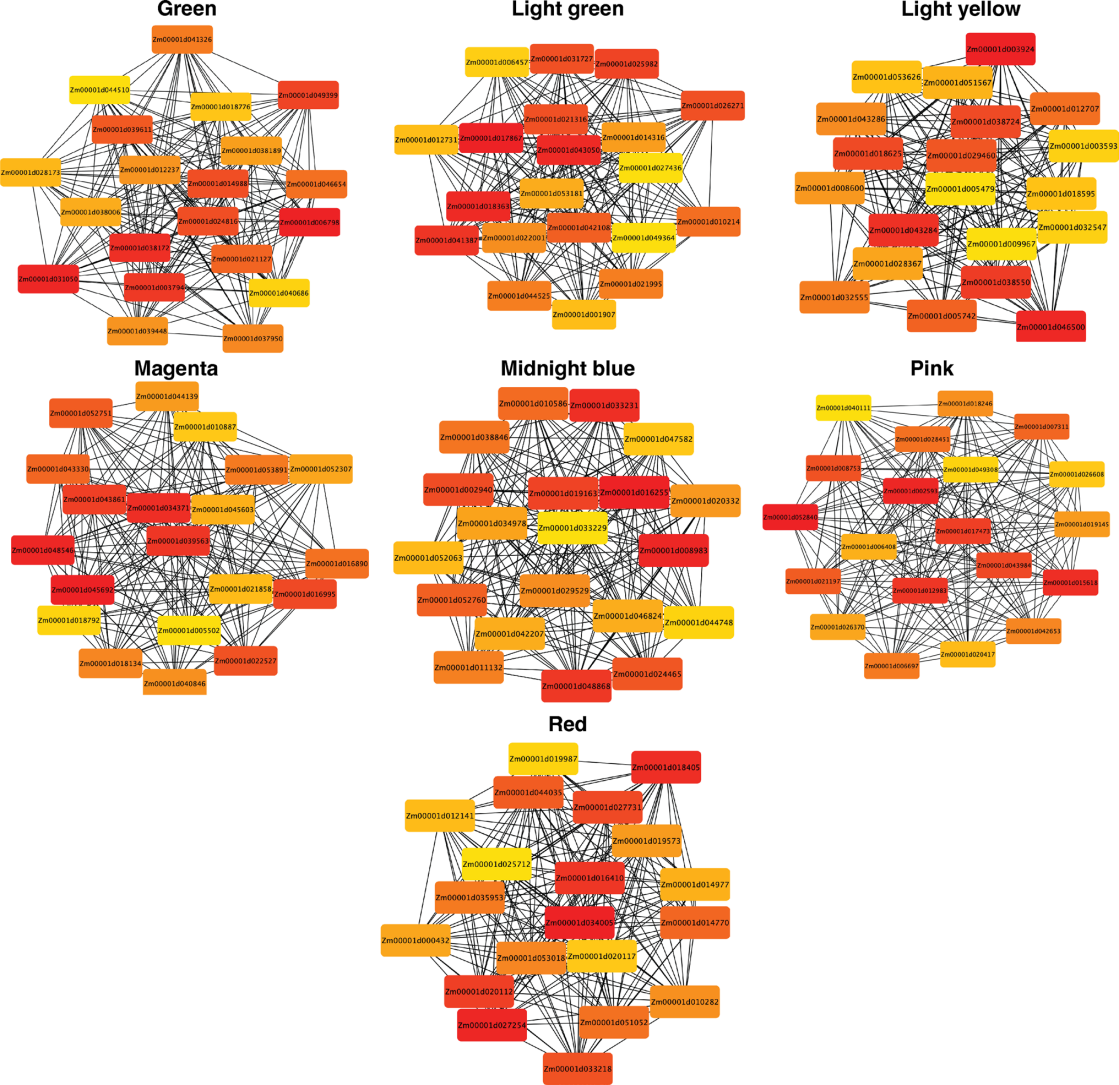
Hub genes identification in seven co-expression modules (CMs)

**Fig. 8:**
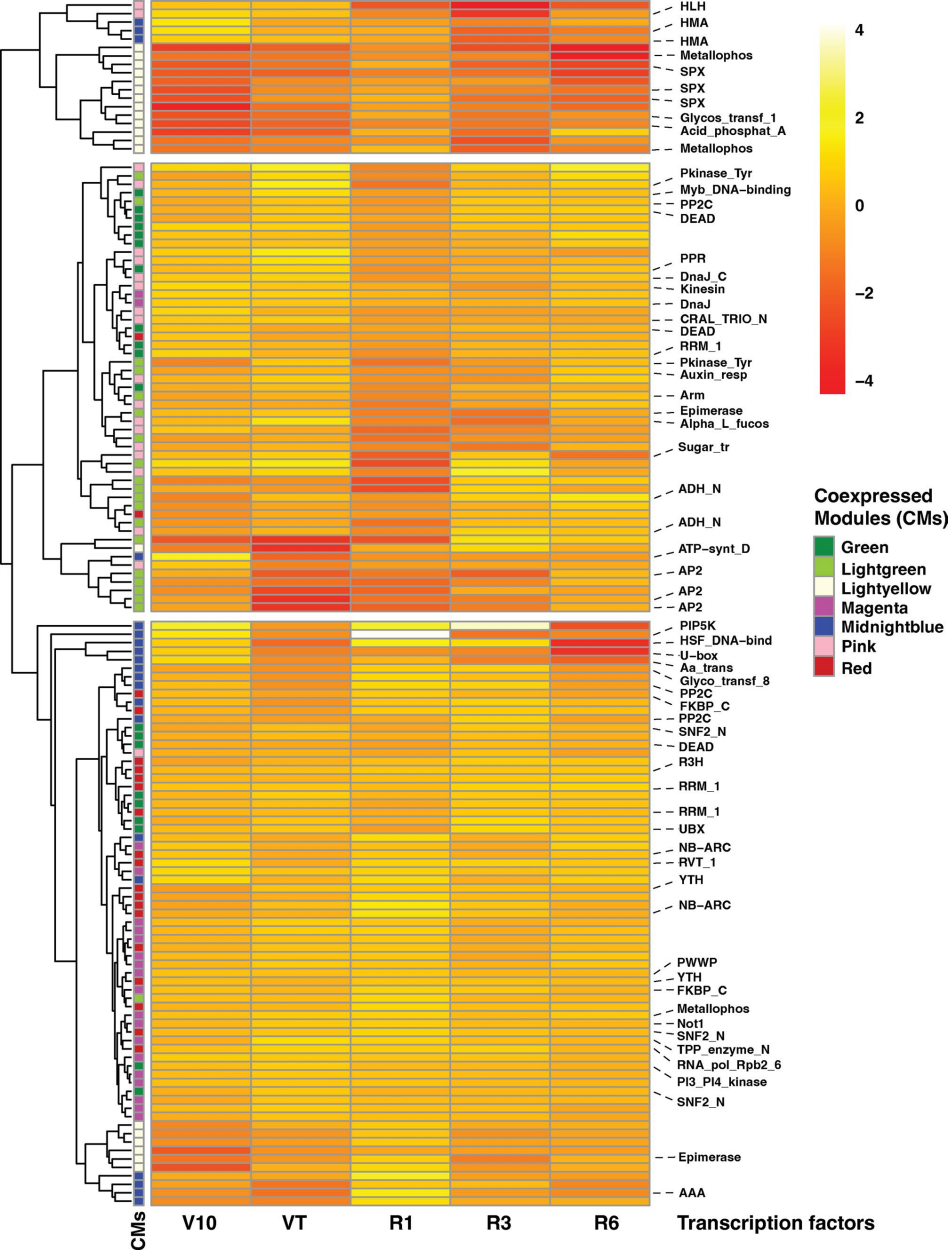
Heatmap of expression dynamics of hub genes in seven co-expression modules (CMs). Z-score was applied for each row. Genes in each CM are associated with a color on the right of the heatmap

Hub genes showed the most connections in the network, as indicated by their high Maximal Clique Centrality (MCC) score (Chin et al. 2014). The top twenty genes with the highest MCC values in each of the specific modules are shown in Fig. 7 and Suppl. Table S7. Among the identified hub genes, many TFs were highly enriched in different developmental stages (Fig. 8). In the green CM, 10 genes encode TFs, including three DEAD-box ATP-dependent RNA helicase, a plant UBX domain-containing protein, a nucleolin-like family protein, a pentatricopeptide repeat-containing protein, two ATP-dependent DNA helicase, an MYB family TF, and a serine/threonine-protein kinase TOR. In light green CM, 8 genes encode TFs, including three ethylene-responsive TFs, an auxin response factor, an armadillo/beta-catenin-like repeat family protein, a receptor-like protein kinase, a sorbitol dehydrogenase, and a protein phosphatase 2C. The light yellow CM hub genes were mainly down-regulated at V10, VT, R3, and R6 stages. Three MYB family TFs, a UDP-sulfoquinovose synthase chloroplastic TF, and a calcineurin-like metallophosphoesterase superfamily protein were identified in the light yellow module. The hub genes in Midnight blue and magenta CMs were more highly up-regulated expressed in the R1 stage. Six and eleven TFs were identified in these two modules, respectively. However, highly down-regulated expression of the hub genes was observed in pink CM at the R1 stage. Nine of 20 hub genes encode TFs, including a BHLH TF, leucine-rich repeat receptor-like tyrosine-protein kinase PXC3, and leucoanthocyanidin reductase. In the red CM, hub genes were mostly up-regulated expression at different developmental stages, and 11 out of those hub genes encode TFs (Fig. 8).

## Discussion

### Impacts of K deficiency on RSA and grain yield in maize

The root, the primary organ responsible for sensing and absorbing nutrients, exhibits a remarkable level of plasticity in response to changes in nutrient availability. The extent of this plasticity varies depending on factors such as plant species, genotypes, the severity and duration of the deficiency, as well as the developmental stage of the plant. Previous studies have demonstrated that low-K stress-induced root growth inhibition is a common phenomenon shared by most plants (Hermans et al. 2006; Jordan-Meille et al. 2018). For example, K^+^ deficiency was found to inhibit the elongation of the main roots and decrease the number and length of the lateral roots in Arabidopsis (Kellermeier et al. 2014). Similar results have been observed in various crops, including rice, cotton, and maize (Zhang et al. 2009; Ruan et al. 2015). In the present study, we also found K^+^ deficiency significantly inhibited the root growth of maize. Most root traits, including the total root length, primary root length, root width, and maximum root number, were reduced by more than 50% to 80% (Fig. 1). The inhibition of root growth induced by K^+^ deficiency may be attributed to the reduction in carbohydrate transport from the shoot to the root, which is crucial for supporting root growth, as K^+^ is necessary for stimulating H^+^-ATPase, which is required for the proper functioning of sucrose transporters (Sustr et al. 2019).

It is known that sufficient K supply is required for crop production. In a long-term experiment conducted to determine the responses of wheat and rice yield to different potassium levels, the results revealed that without the application of K fertilizer over an extended period, the wheat and rice yields were reduced by 47% and 15%, respectively (Song et al. 2018). A similar trend of severe wheat grain yield reduction was reported following a long-term K deficiency (Zörb et al. 2014). In our study, K deficiency led to a 16.8% (*P* < 0.05) reduction in grain yield (Fig. 2). It has been demonstrated that K supply significant effects crop yield by regulating the photosynthesis rate and driving assimilate transport into grains. Potassium impacts photosynthesis through influences on stomatal functioning. It activates enzymes involved in photosynthesis and helps regulate stomata movement, which is controlled mostly by K ion concentration (Srivastava et al. 2020). There is also evidence that plants experiencing inadequate potassium levels can exhibit an elevated production of reactive oxygen species (ROS) within their photosynthetic tissue, consequently leading to photooxidative damage when exposed to higher light intensities (Cakmak 2005).

### Unveiling the influence of K deficiency on nitrogen and phosphorus uptake in maize

Nutrient interactions are common in higher plants, with one element's deficiency or excess affecting the absorption, distribution or function of another. For instance, K deficiency could inhibit N absorption in cotton and significantly reduce leaf nitrate content (Hu et al. 2017). K deficiency also hindered phosphates and magnesium uptake in plants. In our study, K starvation significantly reduced P levels and had a minor effect on N in maize seeds (Fig. 2). K deficiency can inhibit N accumulation in plants by downregulating root nitrate transporters (Daliparthy et al. 1994). Additionally, the role of potassium in regulating water uptake and transport in plants is crucial. This function may aid in the absorption and utilization of other essential nutrients, including nitrogen and phosphorous (Malvi 2011). Maintaining a balanced supply of nutrients is important for optimal plant growth and crop production. Further molecular studies are needed to better understand how K influences other essential elements' homeostasis.

### Gene expression profiling of maize roots reveals stage-specific responses to K deficiency

The plant response to K deficiency involves the regulation of a large number of genes (Ma et al. 2020). To gain a deeper understanding of the complex molecular regulatory mechanisms controlling maize root response to K deficiency, we used a comparative RNA-Seq-based approach to compare gene expression profiles of maize roots under K deficiency at five-time points during plant development. In this study, we identified multiple differentially expressed genes (DEGs) at various developmental stages (Fig. 4). The varying numbers of genes responding to potassium deficiency at different developmental stages suggest that the specific genes involved in the plant root response to potassium deficiency vary across developmental stages. The V10 stage exhibits the highest number of DEGs, whereas the R6 stage shows the lowest number, indicating that maize roots are more sensitive to potassium deficiency during early developmental stages. In other words, maize may require more potassium during the early developmental stage compared to the later stages, which aligns with our K accumulation data, demonstrating a declining trend throughout plant development.

### The role of K^+^ channels and transporters in plant response to K deficiency

Plants have a wide variety of transport systems that play crucial roles in K^+^ uptake, redistribution, and homeostasis. Among them, K^+^ absorption and transport are facilitated by various channels and transporters, with K^+^ channels primarily responsible for mediating low-affinity K^+^ uptake, while K^+^ transporters predominantly facilitate high-affinity K^+^ uptake (Hafsi et al. 2014). It was well documented that many high-affinity K^+^ transporter genes from different plant species are induced by K deficiency, such as AtHAK5, HvHAK1, and OsHAK1 (Gierth et al. 2005; Fulgenzi et al. 2008; Okada et al. 2018). Moreover, a transcriptome analysis of maize roots response to K^+^ starvation revealed that several K^+^ channel genes, including potassium channel KAT1 and inward rectifying potassium channel ZMK1were markedly up-regulated (Zhou et al. 2022). In the present study, consistent with previous reports, a POTASSIUM CHANNEL PROTEIN (Zm00001d016160) was notably induced in response to K starvation at various developmental stages, and HAK5 (Zm00001d033067) was up-regulated upon K deprivation at later developmental stages (Table 1). The findings presented above show that increase in expression levels of genes encoding K^+^ transporters and channels may be a rapid and common regulatory strategy shared by different plant species to increase K^+^ uptake and overcome K^+^ deficiency.

### Transcription factors as key players in maize root response to K deficiency

Transcription factors (TFs) are important regulatory nodes in the regulatory network of plant K deficiency stress response. ARF2 (Auxin Response Factor 2) is well-characterized TF responsible for the negative regulation of AtHAK5 expression. Under K^+^ sufficient conditions, *AtARF2* binds to the *AtHAK5* promoter and represses its transcription. However, in response to low K^+^ stress, *AtARF2* undergoes rapid phosphorylation, leading to its dissociation from the AtHAK5 promoter and subsequent relief of repression on HAK5 transcription (Zhao et al. 2016). An AP2/ERF transcription factor RAP2.11 was identified as a component in response to low potassium through regulation of the high-affinity K^+^ uptake transporter AtHAK5 and other components of the low-potassium signal transduction pathway (Kim et al. 2012).

Other TFs, including *AtbHLH121*, *AtMYB59*, and *OsMYBc,* have been demonstrated to bind to the promoters of high-affinity K^+^ transporters and activate gene expression under K^+^ limiting conditions (Hong et al. 2013; Wang et al. 2015). In this study, *ARF3* (Zm00001d012731) was identified as one of the hub genes, exhibiting significant downregulation across all developmental stages. Interestingly, the maize homolog of *RAP2.11*, Zm00001d030862, displayed downregulation in the root tissue during the early developmental stage (Fig. 8). These findings indicate that while maize roots share some common regulatory strategies with other plants, they exhibit distinct differences in the adaption of K starvation, highlighting the complexity of gene regulation in maize root response to K deficiency. Numerous transcriptome studies have recently reported the differential expression of dozens to hundreds of TFs in plants in response to K deficiency. The MYB, ERF, and WRKY families have emerged as the most frequently identified TFs (Zhao et al. 2018; Yang et al. 2020, 2021).

Consistently, we also found many transcription factors, including MYB, bHLH, ERF, and WRKY families, were highly enriched in different developmental stages response to K starvation (Fig. 8). These results suggest that in addition to the well-established TFs, a multitude of TFs within the MYB, ERF, and WRKY families are implicated in the adaptation to external K^+^ deprivation. However, additional molecular studies are required to validate and further elucidate their specific functions.

### Insights into K^+^ sensing and regulatory mechanisms in maize root response to potassium deficiency

At the cellular level, K^+^ deficiency has been demonstrated to elevate cytoplasmic Ca^2+^ levels that activate downstream signaling through a well-explored CBL (calcineurin B-like protein)-CIPK module (Wang and Wu 2010). Within this module, CBL1/CBL9 interacts with CIPK23 and targets it to the plasma membrane of root cells to phosphorylate a voltage-gated high-affinity K^+^ channel, AKT1 (Arabidopsis K^+^ transporter 1). The phosphorylation of AKT1 enhances its K^+^ uptake activity (Xu et al. 2006). In our study, CALCINEURIN B-LIKE INTERACTING PROTEIN KINASE 23 (Zm00001d018799), CALCINEURIN B-LIKE PROTEIN 10 (Zm00001d044285) were observed up-regulated in specific developmental stage under LK conditions, suggesting that K^+^ sensing and signaling pathway was activated upon maize roots subject to K deficiency (Table 1).

A member of the type III peroxidase family, Rare Cold Inducible gene 3 (RCI3), was identified as a component of the low K signal transduction pathway in Arabidopsis roots. RCI3 is known to be induced in response to K deprivation and is involved in activating of the high-affinity K^+^ uptake transporter HAK5, thereby facilitating K acquisition during K starvation (Kim et al. 2010). Similarly, in our study, the homologous gene of RCI3, Zm00001d014467, was found to be up-regulated in V10 and R1 stages in response to low K conditions, indicating a possible involvement of RCI3 in the early response of maize root to K starvation.

Phosphorylation and dephosphorylation may be important regulatory mechanisms in the plant responses to K deficiency. In the present study, several kinase genes were transcriptionally regulated by K deficiency. Among them, an important serine/threonine-protein kinase TOR (Target of rapamycin) was in the hub gene list (Fig. 8). TOR proteins are members of the phosphatidylinositol kinase-related kinase (PIKK) family, which are highly conserved among yeasts, plants, and mammals. In plants, TOR signaling is a central hub that integrates diverse signals, including nutrients, energy levels, hormones, and environmental cues (Fu et al. 2020). Increasing evidence demonstrated that TOR is involved in plant nutrient deficiency response, such as N, P and S (Dobrenel et al. 2021; Liu et al. 2021; Dong et al. 2022). The identification of TOR as one of the hub genes within the maize root response to K deficiency in our study suggests its potential involvement in the plant's response to K deficiency. However, further research is required to elucidate the specific functions of TOR in this process.

## Conclusion

In summary, our comprehensive analysis of root phenotypes and transcriptome data from five key developmental stages has successfully identified important root system architectures and gene regulatory networks that are implicated in both root development and the response to potassium deficiency in maize. Additionally, we have identified a diverse set of TFs and potential hub genes that are closely associated with the response of roots to potassium deficiency. Interestingly, some of these genes exhibited homology to well-known regulators of root architecture or development in Arabidopsis, such as Zm00001d014467 (*AtRCI3*), Zm00001d011237 (*AtWRKY9*), and Zm00001d030862 (*AtAP2/ERF*). These findings provide a valuable resource for future functional experiments, enriching our comprehension of how root system architecture influences nutrient uptake efficiency and contributes to the continued enhancement of maize grain yield under limiting nutrient availability environments.

### Supplementary Information

Below is the link to the electronic supplementary material.

Supplementary file1 (TIF 47994 KB)

Supplementary file2 (XLSX 64 KB)

## Data Availability

All data generated or analyzed during this study are available from the corresponding author upon request.

## Abbreviations

CMs: Co-expression modules
DEG: Differentially expressed gene
GO: Gene ontology
KEGG: Kyoto Encyclopedia of Genes and Genomes
LK: Low K condition
RSA: Root system architecture
SK: Sufficient K condition
TF: Transcription factor
WGCNA: Weighted correlation network analysis
