# Supplementary figures and images for: Dynamic transcriptome analysis unravels key regulatory genes of maize root growth and development in response to potassium deficiency
Source: Planta. 2023 Oct 14;258(5):99. doi: 10.1007/s00425-023-04260-7 (PMC10576708; doi:10.1007/s00425-023-04260-7)

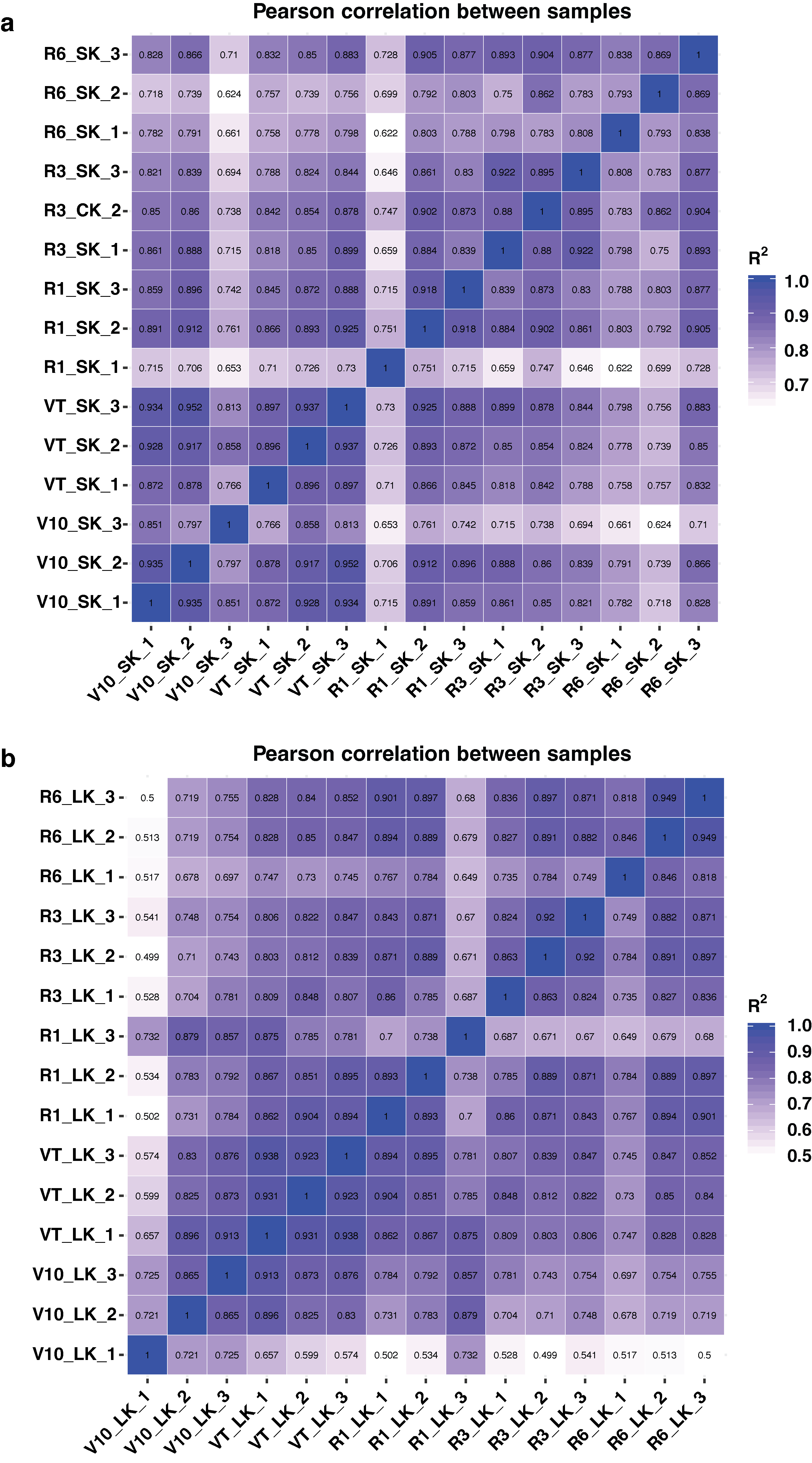

Supplement: Supplementary file 1 — Supplementary file1 (TIF 47994 KB) [file 425_2023_4260_MOESM1_ESM.tif]
